# Battery-free wireless imaging of underwater environments

**DOI:** 10.1038/s41467-022-33223-x

**Published:** 2022-09-26

**Authors:** Sayed Saad Afzal, Waleed Akbar, Osvy Rodriguez, Mario Doumet, Unsoo Ha, Reza Ghaffarivardavagh, Fadel Adib

**Affiliations:** 1 Department of Electrical Engineering and Computer Science, Massachusetts Institute of Technology, Cambridge, MA 02139 USA; 2 MIT Media Lab, Massachusetts Institute of Technology, Cambridge, MA 02139 USA; 3 Program in Media Arts and Sciences, Massachusetts Institute of Technology, Cambridge, MA 02139 USA; 4 MIT Sea Grant, Massachusetts Institute of Technology, Cambridge, MA 02139 USA

**Keywords:** Electrical and electronic engineering, Ocean sciences, Acoustics, Energy harvesting, Imaging and sensing

## Abstract

Imaging underwater environments is of great importance to marine sciences, sustainability, climatology, defense, robotics, geology, space exploration, and food security. Despite advances in underwater imaging, most of the ocean and marine organisms remain unobserved and undiscovered. Existing methods for underwater imaging are unsuitable for scalable, long-term, in situ observations because they require tethering for power and communication. Here we describe underwater backscatter imaging, a method for scalable, real-time wireless imaging of underwater environments using fully-submerged battery-free cameras. The cameras power up from harvested acoustic energy, capture color images using ultra-low-power active illumination and a monochrome image sensor, and communicate wirelessly at net-zero-power via acoustic backscatter. We demonstrate wireless battery-free imaging of animals, plants, pollutants, and localization tags in enclosed and open-water environments. The method’s self-sustaining nature makes it desirable for massive, continuous, and long-term ocean deployments with many applications including marine life discovery, submarine surveillance, and underwater climate change monitoring.

## Abstract (web summary)

The authors present an approach to underwater imaging, which does not require tethering or batteries. The low-power camera uses power from harvested acoustic energy and communicates colour images wirelessly via acoustic backscatter.

## Introduction

Underwater images of marine animals, plants, oceanic basins, coral reefs, and marine debris are key to understanding marine environments and their impact on the global climate system^1–4^. Underwater imaging enables the discovery of new marine species and advances our understanding of the impact of climate change and human activity on the underwater world^1,5,6^. Underwater imaging also supports global aquaculture food production, the world’s fastest-growing food sector, where it is used to detect diseases such as sea lice, monitor harmful algae blooms, and regulate fish feeding patterns to optimize growth^7,8^. More generally, underwater imaging has a large number of applications across oceanography, marine biology, underwater archeology, climatology, space exploration, sustainability, robotics, and defense^9–16^.

Despite advances in underwater imaging, studies estimate that most of the ocean and marine organisms have not been observed yet^17–19^. A long-standing impediment for underwater observations stems from the difficulty of long-term, real-time, in situ imaging of underwater environments. Existing methods for continuous underwater imaging need to be tethered to ships, underwater drones, or power plants for power and communication^6,20–23^. In the absence of such tethering, they rely on batteries which inherently limit their lifetime (and require expensive oceanographic missions for battery replacement). In principle, one could overcome this limitation and power up underwater cameras by harvesting energy from ocean waves, underwater currents, thermal gradients, or sunlight^23–27^. However, adding a tidal, solar, or wave harvester to each underwater camera would make it significantly more bulky and expensive, and may limit its deployment environment (for example, solar and wave harvesters work well only near the surface). As a result, it remains challenging today to perform sustainable, continuous, and distributed underwater imaging.

Here, we report underwater backscatter imaging, a battery-free wireless imaging method for underwater environments. Our method consumes five orders of magnitude less power than previously reported underwater wireless imaging systems^28–30^. The ultra-low-power nature of our method enables it to operate entirely based on harvested energy. Independence of batteries enables long-term, in situ imaging of remote underwater objects, and wireless communication enables real-time monitoring of underwater environments. As a result, this method may be deployed at scale to discover rare species and observe marine populations, act as early warning systems for diseases in aquaculture farms, monitor geological processes (such as submarine volcanoes) and changes in ocean currents, and more closely surveil commercial and military operations^17,22,31^.

## Results and discussion

### Wireless imaging method design and architecture

Our method encompasses fully-integrated ultra-low-power operations including optical sensing, active illumination, processing, and wireless communication. It is capable of performing passive imaging as well as active color imaging using ultra-low-power active illumination, which enables it to operate in different lighting conditions, including complete darkness. Captured images are communicated to a remote receiver that uses them to reconstruct color images of underwater environments. This method can be powered by energy harvested from external sources, such as acoustic, solar, thermal, or ocean current energy. We implement acoustic energy harvesting because of its high efficiency, low cost, and capacity for long-range propagation in underwater environments^32^. The same approaches to energy-neutral imaging can be realized with other sources of ambient energy, such as solar, thermal, or ocean current energy.

**Fig. 1:**
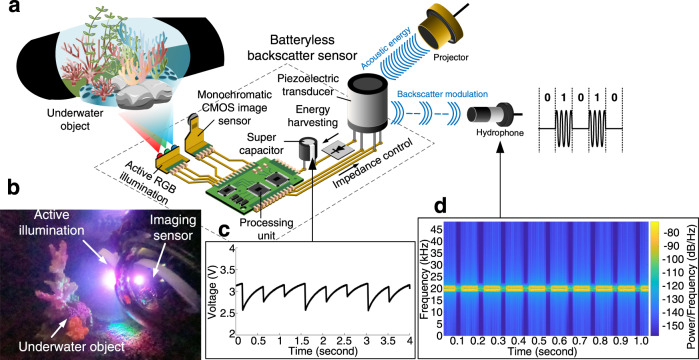
Overview of underwater backscatter imaging. **a** A remote acoustic projector (top right) transmits sound on the downlink. The acoustic energy is harvested by a piezoelectric transducer and converted to electrical energy that powers up the batteryless backscatter sensor node. The energy accumulates in a super-capacitor that powers up an FPGA unit, a monochromatic CMOS sensor that captures an image, and three LEDs which enable RGB active illumination. The captured image is communicated via acoustic backscatter modulation on the uplink, and a remote hydrophone measures the reflection patterns to reconstruct the transmitted image. **b** The batteryless sensor is shown in an experimental trial where it is used to image an underwater object with active illumination that enables capturing color images. **c** The plot shows the voltage in the supercapacitor, which is harvested from acoustic energy and varies over time as a function of the power consumption of different processing stages. **d** The spectrogram shows the frequency response of the signal received by the hydrophone over time, demonstrating its ability to capture reflection patterns due to backscatter modulation and decode them into binary to recover the transmitted image.

Figure 1a schematically summarizes the key components of this wireless imaging method. In acoustically-powered underwater backscatter imaging, a remote projector transmits an acoustic signal on the downlink. Our battery-free sensor node harvests energy from the received acoustic signal using piezoelectric transducers. The received acoustic energy is converted to electrical energy, rectified using a full-wave rectifier, and stored in a super-capacitor. When the stored energy reaches a minimum required threshold, it autonomously activates a power management unit to regulate the voltage and supply it to an on-board processing and memory unit (realizable as a field-programmable gate array or FPGA) and ultra-low-power oscillators. The processing unit and oscillator trigger an ultra-low-power monochromatic CMOS camera and on-board active illumination to capture the image of an underwater object (Fig. 1b). The entire imaging process is powered by the harvested energy in the super-capacitor, whose stored voltage varies over time as a function of the power consumption of different processing stages (Fig. 1c).

A critical step toward realizing battery-free imaging is the development of a technique for ultra-low-power underwater communication. Specifically, the communication component of the system must not consume more energy than what can be harvested from the remote acoustic source, which typically ranges from a few tens to hundreds of microwatts (see Range Analysis in Supplementary Information for an analysis of acoustic energy harvesting as a function of distance). However, state-of-the-art low-power underwater communication modems require 50–100 milliwatts to communicate over tens of meters^33^. Thus, they would require three to five orders of magnitude more power than what is available from harvesting. This significant energy imbalance would make battery-free operation with these modems impractical (see Supplementary Discussion in Supplementary Information).

To operate within the energy harvesting constraints of our proposed battery-free imaging method, we leverage piezo-acoustic backscatter to communicate the captured image on the uplink, extending a recently developed net-zero power communication technology^34,35^ to enable telemetry of imaging data. Underwater piezo-acoustic backscatter communicates messages by modulating the reflection coefficient of its piezoelectric transducer (Fig. 1a). Specifically, due to the electromechanical coupling between a piezoelectric transducer and its electrical impedance load, it is possible to modulate the transducer’s radar cross section. Thus, the battery-free node encodes pixels into communication packets by switching between different electric loads (inductors) connected to the transducer (see “Communication through backscatter” in Methods). The switching is done by simply controlling two transistors and is realizable with 24 nanowatts of power. A remote hydrophone measures the received acoustic signal to sense changes in the reflection patterns due to backscatter (Fig. 1d). The reflection patterns are decoded and used to reconstruct the image captured by the remote battery-free cameras. Robust end-to-end communication is realizable by implementing a full networking and communication stack that incorporates underwater channel estimation, packetization, and error detection (see “Uplink decoding” in Methods).

**Fig. 2:**
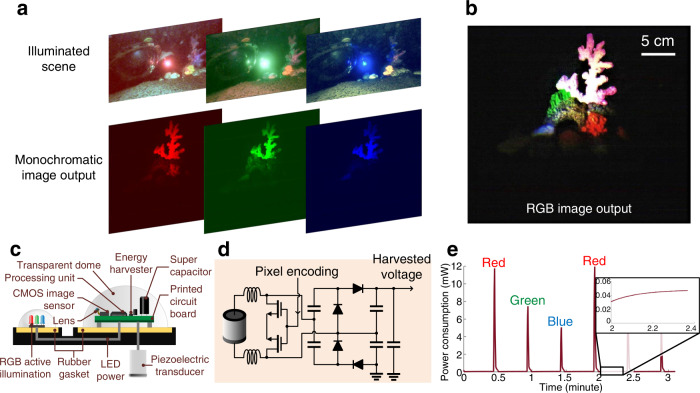
Active illumination in underwater backscatter imaging. **a** To recover color images with a monochrome sensor, the camera alternates between activating three LEDs—red, green, and blue. The top figures show the illuminated scene, while the bottom figures show the corresponding captured monochromatic images, which are transmitted to a remote receiver. **b** The figure shows the color image output synthesized by the receiver using multi-illumination pixels which are constructed by combining the monochromatic image output for each of the three active illumination LEDs. **c** A side view of the camera prototype demonstrates a larger dome which houses the CMOS image sensor and a smaller dome which contains the RGB LEDs for active illumination. The structure is connected to a piezoelectric transducer. **d** The circuit schematic demonstrates how the imaging method operates at net-zero power by harvesting acoustic energy and communicating via backscatter modulation. **e** The plots show the power consumption over time. The power consumption peaks during active imaging and drops when the captured images are being backscattered.

Our method is capable of capturing color images of underwater objects at ultra-low power even in low-lighting conditions, which are standard in the deep sea due to light absorption in the water column. To do so, we utilize an ultra-low-power CMOS imaging sensor (HM01B0 from Himax Corporation), which can capture monochromatic images. To reconstruct color images using the monochromatic imaging sensor, we devised a method for low-power multi-color active illumination. Our battery-free imaging system incorporates three monochrome light-emitting diodes (LEDs): red, green, and blue. An ultra-low-power processing and memory unit (IGLOO nano FPGA) alternates between activating each of these LEDs and captures monochromatic images with each active illumination cycle (Fig. 2a). The monochromatic images are acoustically backscattered to the remote receiver. After decoding each of the images, the receiver synthesizes the received packets into multi-illumination pixels by applying them to the RGB channels of a digital pixel array to reconstruct color images, demonstrating the possibility to recover color patterns of underwater objects such as corals (Fig. 2b, see Supplementary Movie 1).

We demonstrate that in situ underwater wireless batteryless imaging is possible using a self-powered camera system (Fig. 2c) that harvests acoustic energy and communicates using piezo-acoustic backscatter (Fig. 2d). The harvested energy is expended in cycles that alternate between imaging and communication (Fig. 2e). Upon capturing image segments, the processing unit packetizes the pixels and communicates them using piezo-acoustic backscatter, at a power consumption of 59 μW. To deal with the bandwidth mismatch between the ultra-low-power CMOS image sensors (few Mbps) and the underwater acoustic communication channel (few kbps), the captured images are buffered in the memory unit cells (see “FPGA control and logic” in Methods). Our fabricated opto-electro-mechanical system consists of multilayer piezo-electric transducers, electronic components (diodes, capacitors, low-power voltage regulators, and DC-DC converters, low-power oscillators), a processing and memory unit (FPGA), LEDs, and a CMOS image sensor (Supplementary Fig. 1). Active illumination using the LEDs is the most power consuming operation of the battery-free imaging system. For acoustic communication rates of 1 kbps, empirical measurements demonstrate an average power consumption of 276.31 μW for active imaging (see “Power analysis” in Methods and Supplementary Table 1). In our demonstrations of passive monochromatic imaging where active illumination is not needed, the batteryless camera consumes an average of 111.98 μW (Supplementary Table 2). In both configurations, the entire energy budget is harvested from underwater acoustics. Other configurations with different throughput and active illumination techniques are possible.

### Experimental demonstration and evaluation

**Fig. 3:**
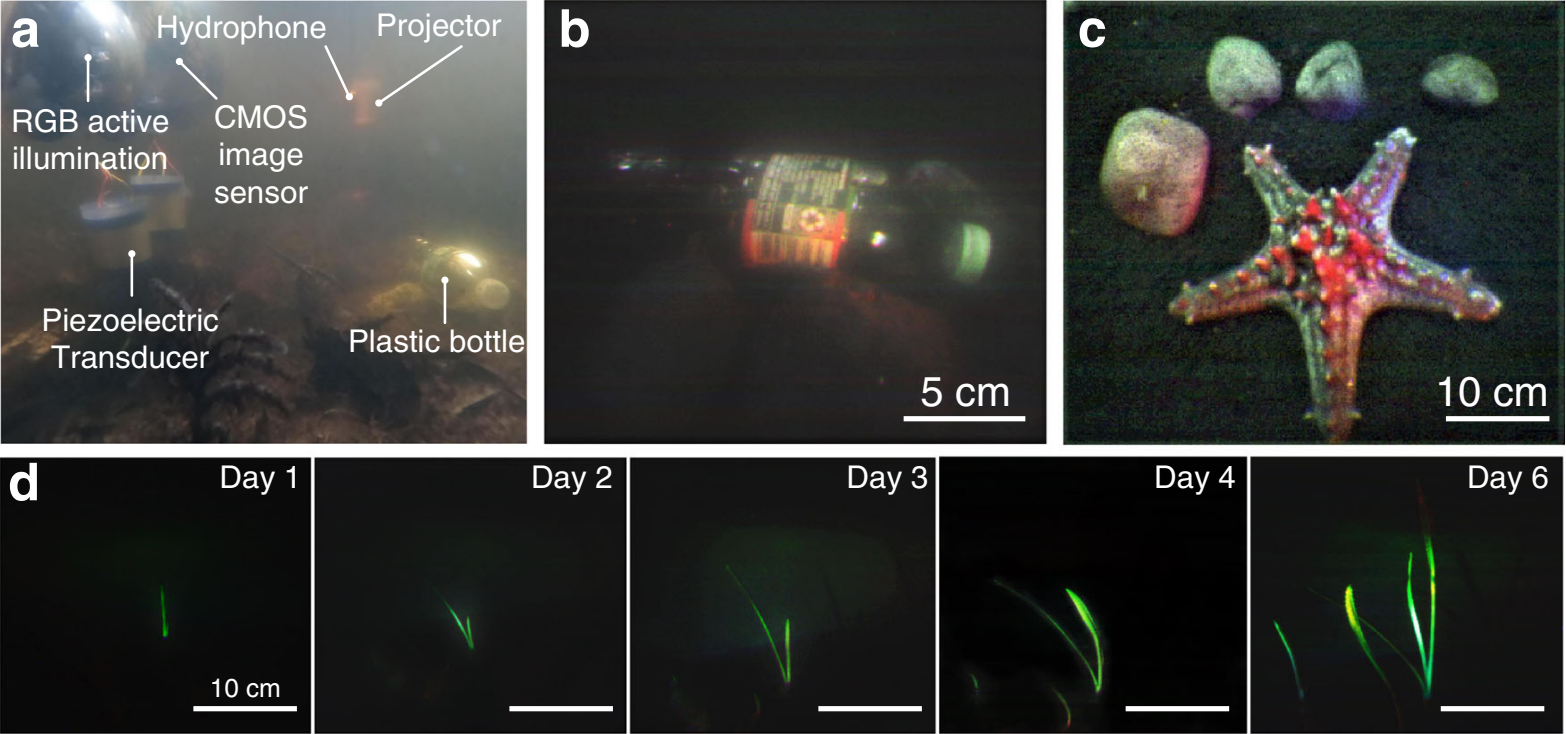
Sample images obtained using underwater backscatter imaging. **a** The figure shows a photo of a prototype deployed in Keyser Pond for monitoring pollution from plastic bottles on the lakebed. **b** The RGB image output obtained from the imaging method while monitoring pollution in Keyser Pond. **c** RGB image output for *Protoreaster linckii*, demonstrating qualitative success in recovering its color and numerous tubercles along the starfish’s five arms. **d** The imaging method was used to monitor the growth of an *Aponogeton ulvaceus* over a week. The figures show the captured images on different days of the week.

We built a proof-of-concept prototype to demonstrate underwater backscatter imaging with animals, plants, and pollution across controlled and uncontrolled environments. The prototype was tested in Keyser Pond in southeastern New Hampshire (43°N, 72°W), where it was used to image pollution from plastic bottles on a lakebed at 50 cm from the imaging sensor (Fig. 3a). Here, color imaging using a monochromatic sensor was successful (Fig. 3b), despite the presence of external illumination. The prototype was also successful in imaging the *Protoreaster linckii*, also known as the African starfish, in a controlled environment with external illumination; the captured image displays numerous tubercles along the starfish’s five arms (Fig. 3c). Furthermore, due to the ability of underwater backscatter imaging to operate continuously, the method was successful in monitoring the growth of an *Aponogeton ulvaceus*, where imaging was performed in the dark over a week, while relying entirely using the harvested energy and active multi-color illumination (Fig. 3d). In all of these scenarios, theprototype was fully-submerged, wireless, batteryless, and autonomous.

**Fig. 4:**
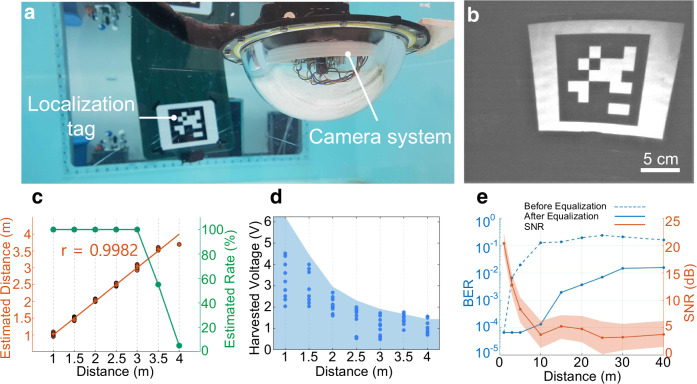
Captured images of AprilTag markers demonstrate successful underwater inference and localization. **a** The prototype was used to detect and localize submerged localization tags. **b** An image of the AprilTag obtained using a batteryless prototype. **c** The estimated location of the AprilTag is plotted in red as a function of its actual location, and the detection rate of AprilTag is plotted in green as a function of distance. **d** Harvested voltage is plotted as a function of distance between the transmitter and the batteryless camera prototype. The dots indicate the voltage at depths, while the contour indicates the maximum voltage obtained when the node’s depth is varied over the entire water column at the corresponding distance. **e** SNR and BER of the imaging method are plotted as a function of distance. The lower and upper bound of the orange band around the SNR plot indicate the 10th and 90th percentile of the collected SNR data at the corresponding distance. The dotted and solid lines show the BER of the imaging method before and after equalization, respectively.

The benefits of underwater backscatter imaging extend beyond observational monitoring to more complex tasks such as underwater localization and inference. To demonstrate the feasibility of such tasks, the imaging method was used to detect and localize visual tags such as AprilTags (Fig. 4a); these tags have been previously utilized for underwater localization and robotic manipulation^36,37^. Figure 4b shows an image of an AprilTag obtained using underwater backscatter imaging. Figure 4c shows the detection accuracy and the localization distance of the AprilTags imaged at different ranges. The results demonstrate very high detection rate and high localization accuracy (localization error below 10 cm) up to 3.5 m. Beyond this range, the current resolution of the CMOS imaging sensor limits both detection and localization; longer detection ranges would be possible with higher-resolution sensors.

We also evaluated the method’s harvesting and communication capabilities as a function of distance in the Charles River in eastern Massachusetts (at 42°N, 71°W). Figure 4d shows the harvested voltage in the river as the distance between the projector and the batteryless sensor increases. The figure shows that the harvested voltage decreases with distance, as expected. We also tested the method’s ability to communicate with a hydrophone receiver at different distances, and computed the signal-to-noise ratio (SNR) and the bit error rate (BER) of the decoded packets at different distances (Fig. 4e). The plot shows that SNR decays and the BER increases with distance, demonstrating the ability to robustly decode packets beyond 40 m by leveraging a decision feedback equalizer (DFE) at the receiver^38^. These results show that underwater backscatter imaging is a viable batteryless telemetry method and that higher ranges may be realizable with higher levels of underwater acoustics or by leveraging underwater transducers with higher efficiency^39^ (see Range Analysis in Supplemental Information).

In summary, we have demonstrated that wireless battery-free imaging in underwater environments is possible. Our method encompasses a highly efficient underwater color camera and innovations that enable robust acoustic backscatter communication in practical underwater environments. The tetherless, inexpensive, and fully-integrated nature of our method makes it a desirable approach for massive ocean deployments. Scaling the method for large-scale deployments requires more sophisticated underwater transducers or high-power underwater acoustic transmissions. Its scalability may be further enhanced by leveraging a mesh network of buoys like those already being deployed on the ocean surface, networks of subsea robots like Argo floats, or surface vehicles like ships to remotely power the energy-harvesting cameras^40,41^. Massive deployments would enable tracking undersea movements—including the flow of particulate organic carbon^42^, marine animals, and naval assets—at scales not realizable today. These may be used to create more accurate models capable of monitoring climate change^43^, decrease the stealthiness of nuclear submarines through large-scale observations, and advance various marine scientific fields.

## Methods

### Communication through backscatter

To enable ultra-low-power communication, our batteryless sensor employs piezo-acoustic backscatter^34^. Piezo-acoustic backscatter differs from traditional underwater acoustic communication in that it does not need to generate its own acoustic signal to communicate. Instead, it communicates by modulating the reflections of incident underwater sound, and a remote receiver can decode the transmitted data by recovering patterns in the reflected signals.

To transmit the stored image data via piezo-acoustic backscatter, our prototype uses two N-channel MOSFETs to modulate the impedance across the terminals of an underwater transducer. The design uses these MOSFETs to switch the transducer’s reflectivity between two states similar to prior underwater backscatter designs^34,35,44^. The signal-to-noise ratio (SNR) at the receiver is maximized when the complex-valued difference (i.e., amplitude and phase) between the two reflective states is maximum. Through our empirical analysis, we have observed that a high SNR on the uplink channel is achieved when the node switches between an inductively matched load and an open circuit. Hence, the FPGA controls the switch to alternate the load between an open circuit and the inductive load to send image data using bi-phase space encoding modulation (also known as FM0) which is known to have high noise resilience in time-varying channels^45^. Other modulation and coding schemes are also possible.

### Uplink decoding

The backscatter communication signal is received by the hydrophone and decoded using a robust demodulation and decoding pipeline (Supplementary Fig. 2) that is implemented through offline packet processing.

The demodulation pipeline consists of a series of filters followed by a maximum likelihood decoder. To remove noise from the received signal, we use a bandpass filter centered around the carrier frequency of 20 kHz with a passband of 10 kHz from 15 to 25 kHz (the filter is implemented as a linear phase type 1 discrete-time FIR filter with filter length of 297). After the bandpass filter, we downconvert the passband signal to baseband by multiplying it with the carrier frequency (20 kHz sinusoid), then use a low pass filter with a bandwidth of 4 kHz (a linear phase type 1 discrete-time FIR filter with filter length of 347, and 6 kHz stopband frequency) to remove high-frequency components from the signal. To mitigate low-frequency interference from naturally-occurring surface waves and turbulence, we implement a high-pass filter (a linear phase type 1 discrete-time FIR filter with filter length of 4535; the respective passband and stopband frequencies of the filter are 150 Hz and 20 Hz). These filtering stages enable the receiver to operate correctly in uncontrolled and time-varying underwater environments.

After filtering and demodulation, the receiver proceeds to packet detection. Each backscatter packet starts with a preamble, and each image segment is sent over multiple packets (as discussed in the subsequent section on FPGA control and logic). The receiver correlates the raw received signal with a known preamble sequence to detect the beginning of the packet. After packet detection, the receiver proceeds to decoding the FM0-encoded packets in baseband. We implemented a bit-by-bit maximum likelihood decoder that has high resilience to channel variations. Formally, consider a received FM0 symbol of size *n* **x** = x_0_, x_1_,…, x_*n*-1_. The decoding operation is done in two steps. The first step performs mean subtraction, exploiting the fact that each FM0 encoded bit has zero mean with respect to neighboring half bits. Mean subtraction removes the constant self-interference signal from the projector as well as any hardware offsets at the receiver. The mean-subtracted symbol **x**′ can be expressed as:

1 $${{{{{{\bf{x}}}}}}}^{\prime}={{{{{\bf{x}}}}}}-\frac{1}{2n}{\sum }_{i=-\frac{n}{2}}^{n+\frac{n}{2}-1}{x}_{i}$$

The second step is maximum likelihood decoding, which is performed by projecting the mean-subtracted received symbol on the time-series symbols **y**^**0**^ and **y**^**1**^, which represent bits ‘0’ and ‘1’, respectively, as per the equation:

2 $$b=\mathop{{{\arg }}\,{{\max }}}\limits_{k={{{{\mathrm{0,1}}}}}}\left({\sum }_{i=0}^{n-1}\frac{{y}_{i}^{k}x^{\prime}_i}{{{||}{{{{{\bf{x}}}}}}^{\prime} {||}}^{2}}\right)$$

Once a packet is decoded, a packet sequence number is used to identify if any of the packets were missed or dropped during the communication process, and a parity bit helps identify incorrectly decoded packet payloads. The packet number and parity check allow the receiver to detect corrupted or missed packets. By incorporating downlink communication, future designs may leverage this capability to request retransmissions from the batteryless sensor.

Finally, it is worth noting that while our implementation focused on uplink communication between one camera sensor and a hydrophone receiver, it is possible to extend to this design with downlink communication, multiple sensor nodes, and multiple receivers; it is also possible to implement other packet sequences with alternate headers that include additional addressing and coding schemes similar to prior work on underwater backscatter^34,35^.

### Energy harvesting and power management

To operate at net-zero power, the batteryless underwater camera sensor may harvest sufficient energy from a remote acoustic source. We use an underwater projector that transmits a 20 kHz sinusoidal acoustic signal (source level 180 dB re 1 µPa @ 1 m) on the downlink. The transmitter uses a layered transducer node to convert the input electrical sinusoidal wave to an acoustic wave.

The transmitted acoustic signal propagates underwater and reaches our batteryless sensor. On the sensor side, a harvesting transducer converts the mechanical vibrations, which are due to pressure changes of the incident acoustic signal, into an electrical sinusoidal signal that can be used to power the circuit. Since the electrical signal produced by our transducer is an alternating current signal, it first needs to be rectified. In our design, the outer layer of the harvesting transducer is directly connected to the harvester circuit; the harvester circuit is composed of an impedance matching network that ensures maximum power transfer efficiency and a four-stage voltage multiplier that rectifies the incoming differential input voltage and quadruples the rectified DC voltage (Supplementary Fig. 1). The rectifier utilizes Schottky diodes with a maximum forward voltage of 350 mV. This rectified voltage is then fed into a super capacitor. The super capacitor’s output voltage is regulated by a 2.8 V Low Dropout (LDO) which drives digital components such as the bank voltage of the FPGA and two external clocks (32 kHz and 4 MHz). The LDO is connected to a DC-DC step down converter, which steps down its 2.8 V to 1.4 V. This allows running the Himax camera and oscillators at their required voltages while running the FPGA core at 1.4 V to minimize power consumption.

In principle, the regulated voltage can be directly used to power up the rest of the sensor electronics and bootstrap the image capture operation and communication. In practice, however, the harvested power may be less than that required to run the electronics for an entire imaging cycle. This is particularly true when the sensor is further away from the projector, leading to a lower harvested power than that required for imaging. In such scenarios, if the capacitor were to provide energy to the rest of the electronic components prematurely, they would drain its energy and abruptly shut down the circuitry before it can capture an image segment.

To ensure that the rest of the circuit does not power up prematurely, our method incorporates a cold-start phase where it harvests energy in its super-capacitor before it powers on the rest of the circuit electronics. To implement this cold-start phase, our design leverages the DC-DC step down converter as a power-gating mechanism (i.e., as a way to buffer energy before providing it to the rest of the circuit), exploiting the fact that the DC-DC converter controls the core voltage of the FPGA logic unit. To do this, our design uses a potential divider to feed a portion of the capacitor voltage to the “enable” pin of the DC-DC step down, so that the DC-DC activates when the capacitor voltage reaches a desired voltage (e.g., 3.2 V in our design). This allows our circuit to harvest energy for a sufficient period of time before it starts operation. In addition, once the DC-DC turns on, it does not turn off until the enable pin voltage falls below a minimum threshold voltage (e.g., 1.4 V in our design). In other words, hysteresis allows the DC-DC to stay active even when the voltage at the enable pin is fluctuating over a wide range. The fluctuation in voltage typically happens due to two main reasons: the first is the variations in harvested energy (from sound) due to the changing underwater channel, and the second is the variation in current draw from various on-board components during different phases of operation (as described in subsequent sections on FPGA control and logic and on power analysis).

Finally, we discuss how the capacitance (*C*) of 7500 μF and minimum threshold value (*V*_thres_) of 3.2 V are determined. Since the camera sensor requires a minimum voltage (*V*_min_) of 2.8 V for reliable operation, the design of the batteryless sensor must ensure that the capacitor voltage remains above *V*_min_ when the camera is operational. Conservatively, the super-capacitor needs to store enough energy to power the circuit for capturing an entire image segment before the energy drawn causes the voltage to drop below *V*_min_; this analysis is conservative since the sensor continues harvesting even during the imaging phase. Mathematically, we can express this energy buffer as:

3 $${{{{{\rm{Energy}}}}}}\; {{{{{\rm{buffer}}}}}}\ge \frac{1}{2}C{V}_{{{{{{{\rm{thres}}}}}}}}^{2}-\frac{1}{2}C{V}_{{{{{{\rm{min }}}}}}}^{2}$$

The energy buffer was determined empirically by measuring the energy required by the camera prototype to capture an image segment using active imaging (5 mJ, see “Power analysis” in Methods). Given that *V*_min_ is 2.8 V, we can select *C* = 7500 μF and *V*_thres_ = 3.2 V to satisfy the above inequality.

Our proof-of-concept implementation employs two separate transducers for harvesting and backscatter communication. In principle, it is possible to use a single transducer—rather than two—for both energy harvesting and backscatter communication, since both transducers are identical. However, doing so would result in less harvested energy; this is because less energy may be harvested in the open-circuit state than in the inductively matched state. Thus, in our prototype implementation, we decouple the communication from the energy harvesting so that both processes can occur simultaneously without either of them reducing the other’s efficiency. Alternate implementations with a single transducer for both harvesting and communication, or with multiple transducers for each of harvesting and communication are possible. The latter is useful for enabling longer-range operation since the combination of multiple transducers can harvest more energy and achieve higher SNR on the uplink (both of which increase with the number of transducers used).

### FPGA control and logic

A key challenge in enabling net-zero power wireless underwater imaging arises from the limited communication bandwidth of underwater acoustic communication, which is typically of the order of few kilobits/s^46^. Due to the limited bandwidth of underwater acoustic channels, the transfer time of underwater images is typically tens of minutes or even hours^47^. In principle, one could keep the CMOS imaging sensor and LED illumination turned on during this period. However, such an approach would be counterproductive since these components consume significantly more power than the rest of the circuit. Here, it is worth noting that higher throughput (thus shorter transfer time) may be realizable using more advanced modulation techniques such as OFDM^48^. However, these techniques require much higher power consumption than underwater piezo-acoustic backscatter^34^.

To enable low-power operation while dealing with the bandwidth constraints of underwater acoustic channels, our method employs an FPGA that operates in two phases: image capture phase (which is power-limited) and backscatter communication phase (which is bandwidth limited). The operation in each of these phases is optimized to minimize overall energy consumption of the underwater backscatter imaging method and enable net-zero operation, as explained below.

#### Image capture phase

Once the super-capacitor has stored sufficient energy from harvesting (e.g., 3.2 V or higher), the voltage at the enable pin of the DC-DC overcomes its threshold, allowing it to power the FPGA core at 1.4 V, as well as the 32 kHz external oscillator. Once the FPGA core is turned on, it initiates its logic sequence to power on the Himax camera sensor along with an external 4 MHz oscillator. The higher frequency clock signal is necessary to operate the camera (which requires at least 3 MHz) and communicate with it over an I2C interface (100–400 kHz).

Once all the onboard components are powered on, the interfacing process starts. The FPGA configures the camera sensor through the I2C communication bus, which enables it to set different parameters on the camera sensor—such as the image resolution, exposure level, and data bits sequence—to enable adapting the image capture to different environmental conditions. In our implementation, the FPGA logic first resets the camera sensor, then sets the image resolution to Quarter Video Graphics Array (QVGA) frame with a resolution of 324 by 244 pixels (each pixel is represented by 8 bits for a total of 632,448 bits per image) and specifies the data transfer protocol to be serial (using a single port and sending the most significant bit first). Finally, the FPGA sets the clock of the camera sensor core to be master clock (MCLK) divided by 8, or more specifically, 4 MHz/8 = 0.5 MHz. To set these parameters, the FPGA uses two I2C connections (SDA, SCL), and it receives all necessary information from three distinct pins on the camera sensor: (1) HSYNC (or line valid), a signal that goes high when a row of the image is being sent and is low otherwise, (2) PCLK (the pixel clock), and (3) DATA0, where the data is transmitted serially. Moreover, the FPGA controls the power to the camera through the power pins (AVDD/IOVDD) (Supplementary Fig. 1). The CMOS imaging sensor sends an acknowledgment after each I2C instruction, indicating the successful execution of the corresponding instruction.

Upon successful I2C communication, the FPGA powers on the red LED, then instructs the camera sensor to capture an image and initiate data transfer process to the FPGA memory. Due to the limited FPGA on-board RAM size (a total of four 4608-bit blocks), only 12 kbs are saved to memory at a time. The RAM is configured as 256-word-deep FIFO, with 48-bit words. After reaching full memory capacity, the FPGA turns off the camera, LED, and 4 MHz oscillator, and switches to the 32 kHz oscillator as it enters the communication phase where it transmits the stored image segment to the receiver.

#### Backscatter communication phase

During the backscatter communication phase, our FPGA uses the lower frequency oscillator of 32 kHz for reading from the memory and transmitting the stored image data because the data rate is limited to 1 kbps due to the narrow bandwidth of underwater acoustic channels. In addition, the low-frequency oscillator allows the FPGA to operate at extremely low power because its dynamic power consumption decreases with the clock frequency. At 32 kHz, most of the power consumption is static (as opposed to dynamic).

The FPGA encodes the image data into packets (Supplementary Fig. 3). Each 77-bit-long packet contains a 16-bit preamble, followed by a 12-bit packet number, 48 bits of data, and a single parity bit at the end. Furthermore, to help the decoder identify packet boundaries, the FPGA introduces a brief silent period (equivalent to the time needed to transmit 23 bits) at the end of each packet. The FPGA converts the data bits into FM0 modulation. The FPGA feeds the FM0 encoded data bits to the gate pin of the two MOSFETs to communicate the image data through backscatter.

After each image segment (stored in memory) is sent, the aforementioned process repeats for the same segment but with a different LED turned on (i.e., green and then followed by blue). Once the same image segment is transmitted and received for all three illuminations (RGB), the FPGA stores the next segment of the image and repeats the same process until an entire image is transmitted. The FPGA also stores the segment index in a designated register and uses a counter to wait for 12000*segment_index clock cycles to store the desired segment to the FIFO memory.

The overall process requires 53 repetitions (53 segments per image, due to memory constraints on the FPGA) for monochromatic images and 159 for active color illumination. For FPGAs with larger memory size, the number of repetitions will be lower.

### Power analysis

Our proof of concept prototype can perform active and passive imaging at an overall average power consumption of 276 μW (Supplementary Table 1) and 112 μW (Supplementary Table 2), respectively. Note that the additional power requirement for active imaging is due to the active illumination, and that the average power is reported over the duration of capturing and transmitting an entire image (i.e., by dividing the total energy consumed across all operation phases by the time to capture and transmit the image).

Our method’s ultra-low power consumption is realizable due to multiple design factors. First is the use of underwater backscatter communication to transmit pixel data. In contrast to traditional underwater acoustic communication technologies, underwater backscatter does not need to generate its own signal; instead, it communicates by modulating the reflection patterns of incident acoustic signals. The process of switching between the two states requires passive switches (e.g., MOSFETs) which consume 24 nanowatts of power, making the communication process extremely low power. Second is the use of low-cost commercially available ultra-low-power FPGAs, which consume as little as 22 μW during certain phases of the operation. Third is the switched dual-oscillator method (of 32 kHz and 4 MHz), which allows minimizing the energy consumption by adapting clocking to different phases of operation. Specifically, in the bandwidth-limited phase—i.e., when the method is constrained by the bandwidth of the underwater acoustic channel, the method switches to the low-frequency oscillator (32 kHz), minimizing the power consumption of the FPGA. On the other hand, in the power-limited phase—i.e., when the method is limited by the power consumption of the CMOS image sensor (0.77–1.1 mW) and LEDs (1.9–8.2 mW), it switches to the high-frequency clock to rapidly complete the pixel transfer to the FPGA and turn off the camera and LEDs during the communication phase. Since the FPGA switches the high-power components off during communication, the supercapacitor can harvest energy and recharge during that phase. The overall power consumption is optimized through a simple, low-cost, power-management unit with a DC-DC converter, low-power LDOs, and resistor dividers as described earlier. The ultra-low power consumption may be further reduced by duty cycling rather than continuous operation.

## Supplementary information

Supplementary Information

Description of Additional Supplementary Files

Supplementary Movie 1

## Data Availability

All relevant data supporting the findings of this study are available within the main article or in the supplementary information.
