# Publisher Correction: Battery-free wireless imaging of underwater environments

**DOI:** 10.1038/s41467-022-33823-7

**Published:** 2022-10-12

**Authors:** Sayed Saad Afzal, Waleed Akbar, Osvy Rodriguez, Mario Doumet, Unsoo Ha, Reza Ghaffarivardavagh, Fadel Adib

**Affiliations:** 1 Department of Electrical Engineering and Computer Science, Massachusetts Institute of Technology, Cambridge, MA 02139 USA; 2 MIT Media Lab, Massachusetts Institute of Technology, Cambridge, MA 02139 USA; 3 Program in Media Arts and Sciences, Massachusetts Institute of Technology, Cambridge, MA 02139 USA; 4 MIT Sea Grant, Massachusetts Institute of Technology, Cambridge, MA 02139 USA

**Keywords:** Electrical and electronic engineering, Ocean sciences, Acoustics, Energy harvesting, Imaging and sensing

**Correction to**: *Nature Communications* 10.1038/s41467-022-33223-x, published online 26 September 2022

The original version of this Article contained an error in Ref. 39, in which the title of the cited work was missing. The complete citation is: Bereketli, A. Interference-free source deployment for coverage in underwater acoustic backscatter networks. *Peer-to-Peer Netw. Appl.* **15**, 1577–1594 (2022). This has been corrected in the PDF and HTML versions of the Article.

