# Supplementary material for: Battery-free wireless imaging of underwater environments
Source: Nat Commun. 2022 Sep 26;13:5546. doi: 10.1038/s41467-022-33223-x (PMC9512789; doi:10.1038/s41467-022-33223-x)
Supplement: Supplementary file 1 — Supplementary Information [file 41467_2022_33223_MOESM1_ESM.pdf]

# Supplementary Information

## Battery-free Wireless Imaging of Underwater Environments

Sayed Saad Afzal†, Waleed Akbar†, Osvy Rodriguez†, Mario Doumet, Unsoo Ha,
Reza Ghaffarivardavagh, Fadel Adib*

†These authors contributed equally to this work
*Corresponding author. Email: fadel@mit.edu

1. Fabrication Methods
2. Evaluation and Testing
   2.1. Enclosed Water Testing Environments
   2.2. Open Water Testing Environments
3. Range Analysis
   3.1. Open-Circuit Voltage
   3.2. Harvested Voltage
   3.3. Harvested Power
4. Timing Analysis
   4.1. Energy Harvesting Time
   4.2. Image Framerate
5. Cost Analysis
6. Supplementary Discussion
   6.1. Comparison to Low-Power Acoustic Modems
   6.2. Comparison to Alternative Underwater Communication Technologies
7. Supplementary Figures
8. Supplementary Tables
9. Supplementary References

## 1. Fabrication Methods

The underwater camera is attached to two piezoelectric transducers. The fabrication process of these transducers is similar to previous methods of building transducer nodes for underwater communication[1,2]. Each underwater transducer contains two different types of piezoceramic cylinders (Supplementary Fig. 4). The outer piezoceramic cylinder has an outer radius of 27mm, inner radius of 23.5mm, height of 40mm, and a nominal resonance frequency of 17 kHz in radial mode (SMC5447T40111, Steminc), while the inner piezoceramic cylinder has an outer radius of 18mm, inner radius of 15.5mm, height of 20mm, and a nominal resonance frequency of 30 kHz in radial mode (SMC3631T20111, Steminc). We stacked two of the inner piezoceramic cylinders and soldered them together to obtain the same height as that of the outer cylinder. We laser cut polyurethane gaskets from an abrasion-resistant polyurethane rubber sheet (40A, McMaster-CARR) and set them on 3D-printed (Creator Pro, Flashforge) end caps, and we tightly screwed the entire structure together to prevent leakage. We placed this entire structure inside a 3D printed cylindrical mold with 3.0 cm radius and 7.5 cm height, and we poured a polyurethane mixture (WC-575A/B, BJB Enterprises) into the mold to insulate it from the surrounding environment. The top and base lids have openings in between the outer and inner cylinders which allow the mixture to fill in the gaps between the cylinders. Afterwards, we placed this structure inside a pressure chamber (Pressure Chamber, Smooth-On) for 12 hours at a pressure of 60 psi to remove residual bubbles from the polyurethane solution. After removing the node from the pressure chamber, we manually removed the mold. We used this procedure to fabricate the transducers for both the projector and the camera. We simulated the beam pattern and directivity of these transducers using COMSOL Multiphysics (COMSOL). The transducers have a toroidal radiation pattern with a directivity index (DI) of 2.62 dB (Supplementary Fig. 8).

The housing of the camera prototype consists of two dome structures (Supplementary Fig. 5). The larger dome is six inches in diameter (6'' Dome Port Lens, TELESIN) and houses the circuitry. The smaller dome comprises of an in-house built plastic base, an abrasion-resistant polyurethane rubber sheet, an acrylic dome which is 3 inches in diameter (Plastic Hemisphere, SupremeTech), and six screws to hold the entire structure together.

The electrical components of the design include a PCB (designed using a freely available software (Eagle, Autodesk) sent for fabrication to a commercial vendor (EasyPCBUSA, Sun Circuits)), an FPGA (IGLOO nano AGLN060, Microsemi), a CMOS monochrome camera sensor (HM01B0, HiMax), a camera connector (609-4320-2-ND, Digikey), two oscillators of 32 kHz (SiT1566AI-JV-18E-32.768E, SiTime) and 4 MHz (SiT8021AI-J4-18S-4.000000E, SiTime) frequencies, a 7500 μF supercapacitor (667-EEU-FS0J752S, Mouser Electronics), a 2.8 V voltage regulator (TPS7A0328PDBVR-LDO, Texas Instruments), a DC-DC step down converter (TPS62841DLCR, Texas Instruments) and red, green, and blue LEDs (604-WP154A4SUREQBFZG, Mouser Electronics). Additional components include four schottky diodes of 0.35 Volt threshold (750-CDBU0130L, Mouser Electronics), four capacitors of 0.1 μF value (587-3502-1-ND, Digikey), three other capacitors of values 47 μF (490-10559-1-ND, Digikey), 10 μF (810-CGA3E1X7T0G106M0, Mouser Electronics), and 4.7 μF (80-C0603C475M9P7411, Mouser Electronics), four resistors of 1 MΩ, two resistors of 4.7 KΩ (13-RE0603FRE074K7LCT-ND, Digikey), one 2.2 μH inductor (118-CC453232A-2R2KLTR-ND, Digikey), and six 750 μH inductors (HM3341-ND, Digikey). Each electrical component is individually tested and manually soldered using a digital hot air rework and soldering station

(AO888A, Aoyue). Power measurements of electrical components were made using a power profiler (PPK2, Nordic Semiconductor).

A function generator (SGD 1032x, Siglent) connected to a fabricated piezoelectric transducer (fabrication procedure described above) through an audio amplifier (XLi 3500, Crown) is used as an underwater projector to transmit acoustic signals. An acoustic hydrophone (H2A, Aquarian) is used as a remote receiver to measure underwater sound. The hydrophone is connected to a laptop (XPS 15 7590, Dell), which records sound using an open-source audio recording software (Audacity) at a sampling rate of 192,000 samples/sec. The signal processing and decoding algorithms are implemented in MATLAB R2020b (Mathworks). The FPGA program is designed using a freely available IDE (Libero SoC v11.9, Microsemi), and the IDE-generated programming file is flashed on the FPGA using a programmer kit (FlashPro 3, Microsemi).

## 2. Evaluation and Testing

The batteryless camera prototype was evaluated qualitatively and quantitatively in enclosed and open water environments.

### 2.1. Enclosed Water Testing Environments

**Imaging:** Testing in controlled environments was performed in an enclosed water tank with a depth of 1.5 m and rectangular cross section of 3 m x 4 m (Supplementary Fig. 6). Here, the projector, hydrophone, and the two transducers of the batteryless camera (for harvesting and backscatter) were all submerged at a depth of 75 cm below the water surface. At the same time, the domes housing the camera and illumination (which are connected to the two transducers using wires) were placed along with the underwater objects in a separate tank to isolate them and control environmental conditions including lighting and nutrient levels. Specifically, the coral reef model and the *Protoreaster linckii* were co-located with the camera at the base of a smaller tank with a depth of 40 cm and a rectangular cross-section of 40 cm x 50 cm (Supplementary Fig. 6). Similarly, several seeds of *Aponogeton ulvaceus* were planted in freshwater aquarium substrate in a third tank with the same dimensions (40 cm x 50 cm x 40 cm), and the camera was used to monitor their growth over a period of one week. Images in Fig. 2b, Fig. 3c, and Fig. 3d of the main text demonstrate successful imaging in these evaluation scenarios.

**AprilTag Data Collection:** Data collection for the AprilTag localization and detection task was performed in the larger tank (3 m x 4 m x 1.5 m). For this task, the camera sensor was submerged in the tank at a depth of 30 cm below the surface and placed at one side of the tank to capture images of the AprilTag. The AprilTag was submerged at the same depth. The experimental trial was repeated by placing the AprilTag at 8 different locations separated by 50 cm, up to 4 m of maximum range between the AprilTag and the camera (i.e., the edge of the enclosed tank). At each location (i.e., range), we used the camera to capture 20 images of the AprilTag, where the orientation and angle was varied with respect to the camera in each image, resulting in a total of 160 images (Supplementary Fig. 7). To speed up the data collection process, these images were collected by connecting the FPGA output directly to a USRP N210 software radio (Ettus); this removes the bandwidth limitation of underwater acoustic communication and enables programming the FPGA to transmit captured pixels at a much higher rate (2 Mbps). Note that we did not bypass the FM0 backscatter modulation for the results shown in Fig. 4c, but only bypassed the underwater channel. In addition to this data collection, Fig. 4b of the main text shows a sample AprilTag image captured in this setup using end-to-end batteryless imaging and underwater backscatter communication (at 1 kbps).

**Calibration for AprilTag Localization:** In order to determine an accurate relationship between a 3D location in the environment and its corresponding 2D pixel in the image captured by our underwater camera, we compute the 3 x 3 homography matrix that contains all the physical information (location and orientation) of the tag[3]. Computation of the matrix requires the intrinsic parameters of the camera, such as the focal length and optical center of the camera. To extract the parameters from our underwater camera, we used a checkerboard calibration method, which is standard in 3D reconstruction problems in computer vision[3]. We captured 150 images of the

checkerboard (7x10 square pixels with a pixel size of 23 mm x 23 mm) from different viewpoints at 3 different distances: 50 cm, 80 cm, and 120 cm and extracted the intrinsic parameters using the Multiplane calibration algorithm[4]. This calibration process needs to be completed only once since we used the same underwater camera throughout all of the measurements.

**AprilTag Detection and Localization:** After the camera is calibrated, the detection and localization tasks are performed on the captured AprilTag images in the dataset described earlier. The tasks were performed following similar procedures to prior work on AprilTag localization[5]. The detection algorithm computes the gradient of every pixel and clusters the pixels that have similar direction and magnitude into components. After performing a recursive depth-first search, it extracts the edges of the AprilTag. Using the edges, the algorithm finds four-sided regions that have a darker interior than their exterior and verifies if the region has valid tag pixels. If the pattern is valid, the detection succeeds, and the region is used as an input to the homography matrix which outputs the tag's location.

## 2.2. Open Water Testing Environments

Open water testing of the prototype was performed in Keyser Pond, NH and in Charles River, MA (Supplementary Fig. 6a, Fig. 6b).

In Keyser Pond, the acoustic transmitter*, harvesting and backscatter transducers, and hydrophone were submerged half a meter below the water surface and the camera sensor was placed at a distance of 50 cm from the plastic water bottle. The image was collected at night, yet the prototype was successfully able to capture color features (as shown in Fig. 3b in the main paper) due to its active illumination method.

Long-range communication experiments were performed in the Charles River, where the acoustic projector, harvesting and backscatter transducers, and hydrophone were all submerged at a depth of 2 m below the water surface. The projector and the backscatter transducer were separated by a distance of 50 cm and the hydrophone was moved further away up to 40 m to test communication at different distances. For this experiment, the backscatter node was programmed to communicate a known pseudo-random sequence of 50 bits (10 bits of preamble with 40 bits of data) in each packet at a data rate of 1 kbps. These bits were constructed in MATLAB and were fed to the transistor switches M1 and M2 using a signal generator (Supplementary Fig. S1). The hydrophone was connected to a USRP N210 to record the received signal for 20 seconds, resulting in 400 packets. For each distance, we recorded data at three different depths (1.5 m, 2 m, and 2.5 m), and for each depth, we computed a single value for BER and different values for SNR (one for each decoded packet). The BER value was computed over all packets by comparing the decoded 50 bits of each packet with the actual transmitted bits. SNR values were computed individually for each packet where the signal power was determined by projecting the received packet onto the transmitted packet and noise power was evaluated by subtracting the signal power from the total received power. The SNR and BER curves are shown in Fig.4e of the main text as a function of

---

* In the Keyser Pod experiment, the cumulative sound exposure level ($SEL_{cum}$) value was 191.29 dB re 1 $\mu Pa^2 s$ at a distance of 10 meters from the transmitter. Note that this value is within the limits defined by Marine Mammal Protection Act (MMPA)[6] for all marine mammals except for mammals that lie within high-frequency cetacean hearing group. However, there were no mammals from this group (or any other group) within the 10-meter radius of the transmitter.

distance, where the BER curve shows the median value of BER across all three depths and the solid line for SNR represents the median SNR over 900 packets (300 packets * 3 depths). The lower and upper bound of the shaded region for the SNR curve represent the 10th and 90th percentile respectively.

In addition to testing the communication capabilities of our method, we also evaluated its harvesting performance at different ranges. An experiment was performed in the Charles River, where the acoustic projector[†] and the harvester node were submerged at a depth of 2 m below the water surface. The harvester node was moved further away (with an interval of 50 cm) up to 4 m. The open-circuit, rectified, harvested voltage was measured using a digital oscilloscope. For each distance, the harvester node was moved to three different depths (1.5 m, 2 m, 2.5 m) and the voltage was measured at each depth. At each depth, 3 measurements were taken, resulting in a total of 9 measurements at each range. The harvester node was also moved gradually across the entire water column for each distance to measure the maximum voltage that the harvester transducer can harvest at each distance. The plot for harvested voltage as a function of distance is shown in Fig.4d of the main text where the maximum harvested voltage is represented as the contour of the shaded region and the 9 measurements at 3 different depths are represented as dots.

[†] In our experiments at Charles River, $SEL_{cum}$ value was 168 dB re 1 $\mu Pa^2 s$ at a distance of 10 meters from the transmitter. This $SEL_{cum}$ value is within the MMPA acoustic thresholds for all marine mammal hearing groups.

## 3. Range Analysis

In battery-free backscatter communication systems, the end-to-end communication range is determined by the ability of a remote transmitter to power up the battery-free sensor[7,8]. Hence, to understand the communication range of our underwater battery-free imaging system, we analyze the downlink range between the projector and the battery-free node. Our downlink analysis follows a model introduced in recent work that studied the range of underwater acoustic backscatter communication systems[7].

The downlink communication range of our system is determined by two constraints: (a) the harvested power and (b) the rectified voltage. In particular, the harvested power needs to exceed a minimum threshold for continuous operation, and the rectified voltage needs to exceed a minimum activation voltage required to turn on the LDO (see Energy Harvesting and Power Management in *Methods*). Since the harvested power and the rectified voltage are both a function of the open-circuit voltage, we first analyze the open-circuit voltage as a function of range, then relate it to the harvested voltage and power.

### 3.1. Open-Circuit Voltage

The voltage at the harvesting transducer is a function of the transmit source level (due to transmit power, projector efficiency, and directivity), range and pathloss (due to absorption, spreading loss, and directivity), and the properties of the harvesting transducer (efficiency, directivity and sensitivity). Specifically, the RMS open-circuit voltage ($V_{oc}$) can be expressed as[7,8]:

$$V_{oc} = 10^{\frac{\mathrm{RL}(P_t,f,R)+\mathrm{RVS}}{20}}$$

where RVS is the receiving voltage sensitivity of the backscatter node's transducer, and RL is the received signal level at the transducer, which itself is a function of the transmit power ($P_t$), transmit efficiency ($\eta_{Tx}$), range ($R$), and directivity of the projector ($\mathrm{DI}_{Tx}$), spreading factor ($k$), and absorption coefficient ($\alpha$) as per the following equation[8,9]:

$$\mathrm{RL}(P_t, f, R) = 170.8 + 10\log(\eta_{Tx}P_t) + \mathrm{DI_{Tx}} - k\,.10\log(R) - \alpha(f)R$$

### 3.2. Harvested Voltage

The harvested voltage is a function of the open-circuit voltage ($V_{oc}$). In particular, recall that the harvesting transducer's output (after matching) is passed through a multi-stage rectifier that converts the AC to DC voltage and passively amplifies the voltage. The harvested voltage at output of the rectifier ($V_{rect}$) is a function of the number of stages ($N$) and the diode threshold voltage ($V_{th}$), and can be expressed as follows[10]:

$$V_{rect} = N\left(\sqrt{2}\,V_{oc} - V_{th}\right)$$

In our prototype implementation, RVS = -180dB re 1V/µPa, $\eta_{Tx} = 0.175$, $P_{Tx} = 25$ W, $\mathrm{DI_{Tx}} = 2.62$dB, $k = 1.5$, $\alpha = 0.0022$dB, $N = 4$, and $V_{th} = 0.35$ V.

To study the harvested voltage constraint in our battery-free imaging system, we simulate the rectified voltage as a function of range following the above model (Supplementary Fig. 9a). The figure also plots the minimum activation voltage (dashed horizontal line), which corresponds to 3.2V in our design. We consider three optimizations for our proof-of-concept prototype, following the parameters highlighted in prior work on underwater backscatter[7]. First, we consider a design whose harvesting transducers have an RVS of -157dB re 1V/µPa (instead of -180dB re 1V/µPa), and plot the rectified voltage (in blue). Our second optimization considers a projector whose efficiency is 0.5 (instead of 0.175), and we plot the corresponding rectified voltage (in orange). Finally, we study how increasing the transmit power from 25 W to 500 W impacts the harvested voltage as a function of range (in black). The figure shows that with more optimized engineering parameters, the range of an underwater battery-free imaging system may increase to more than 300 meters, matching prior analytical model[7].

It is worth noting that the activation voltage is also function of our system design parameters. In principle, the main limitation on the voltage is determined by the non-linearity of the harvester electronics, specifically the diodes, whose threshold voltage is 0.35V. One can approach this threshold voltage (and achieve higher ranges) by increasing the number of stages in the multi-stage rectifier as well as by using rectifiers with lower threshold voltages[11].

### 3.3. Harvested Power

Next, we analyze the harvested power as a function of range. The harvested power ($P_{harv}$) is a function of the open-circuit voltage, harvesting circuit efficiency ($\eta_{harv}$), and transducer impedance ($Z$) as per the following equation[7,8]:

$$P_{harv} = \frac{\eta_{harv} {V_{oc}}^2 . \mathrm{Re}(Z)}{\|Z\|^2}$$

In our prototype implementation, $\eta_{harv}$ = 0.16 and $Z$ = 35 - 203j.

We plot the harvested power as a function of range following the same parameters of the above model in (Supplementary Fig. 9b). We also plot the minimum power (dashed horizontal line) required for our prototype to operate continuously. The plot demonstrates that underwater battery-free imaging may be possible at hundreds of meters under optimized engineering design parameters.

It is worth noting that the harvested power can be further improved by optimizing two other design parameters. First, in addition to the parameters discussed above, it is possible to boost the AC-to-DC power conversion efficiency from 0.16 to higher realizable efficiency of 0.60[12]. Second, the end-to-end power transfer efficiency (and range) may be improved by using beamforming[‡]. In particular, past work has considered underwater acoustic beamforming and demonstrated that it can enable directivity gains of 16dB[13]. A natural question here is: how can a projector identify the optimal beamforming direction so that it may electronically steer its array accordingly? If the

---

‡ Note that the current transducer has a toroidal radiation pattern (see Fabrication Methods). Beamforming would allow the projector to focus the energy in a specific direction thereby extending the range of operation for a given input power.

backscatter node's location is known a priori, then the beamsteering direction may be computed geometrically and the projector can apply the corresponding beamsteering vector. Alternatively, if the backscatter node's location (or the projector's location) is unknown, then the projector can find the correct beam by employing one of the standard beam searching algorithms[14,15]. For example, the projector can first scan different directions, by sequentially applying different beamforming vectors. When it reaches the correct direction, the backscatter node powers up and responds with stored bits. The projector uses this feedback to identify the correct direction, and continues beamforming in that direction for the remainder of the communication session. Since the transmit source level in our evaluation is already high (180dB re:1µPa), such optimized designs will be critical to achieve higher range in future work.

## 4. Timing Analysis

In this section, we analyze the timing performance of our ultra-low-power imaging platform. Specifically, we analyze the time that the system needs to harvest sufficient energy to power up and the time needed to capture and communicate one full image.

### 4.1 Energy Harvesting Time

Our battery-free camera sensor operates entirely on the harvested power, and the time, T needed to harvest sufficient energy to capture a gray-scale image is given by the following equation:

$$T = \frac{1.4006 \text{ mJ}}{P_{harv}}$$

where *1.4006* mJ is the energy required during the image capture phase (see Supplementary Table 2) and $P_{harv}$ is the harvested power. The harvested power depends on the transmit power, distance from the projector, harvesting transducer's RVS, and the efficiency of the harvesting circuit. With our current design parameters (see Range Analysis), it takes around 10-12 seconds to harvest sufficient energy at 1 meter. However, recall from our discussion in Range Analysis that these parameters can be optimized to increase the harvested power which would reduce the time needed to harvest sufficient energy. Specifically, using the model parameters mentioned in Range Analysis and the equation given above, we plot the harvesting time, *T* as a function of distance (Supplementary Fig. 9c). The plot shows that under optimized design parameters, the energy harvesting time is less than a second (i.e., the imaging operation starts instantaneously) even beyond 100 meters.

Recall that sending a full image typically requires multiple captures (due to the memory limitations on the FPGA), and one might wonder whether each of these captures requires the above-mentioned harvesting time. However, that is not the case, and the sensor needs the harvesting time only once during the beginning of the operation. To see why, recall that the system operates in two phases: image capture phase and backscatter communication phase. The backscatter communication phase consumes significantly less power of 59 µW (Supplementary Table 2) and lasts longer (due to the narrow bandwidth of the underwater acoustic channel). As a result, the capacitor fully recharges during this phase before it needs to enter the image capture phase again, allowing for uninterrupted operation after the initial harvesting cycle.

Finally, it is worth noting that the above analysis assumes that the system is operating in warm start (i.e., there is some pre-stored charge across the capacitor). During the cold-start phase (which occurs only once in the system's lifetime), the capacitor is fully discharged and the time required to harvest sufficient energy to initiate the operation is given by:

$$T = \frac{\frac{1}{2} C\, {V_{thres}}^2}{P_{harv}}$$

Where *C* is the capacitance value (7500 µF) and $V_{thres}$ is the threshold voltage (3.2 V) across the capacitor needed to initiate the operation. With our current design parameters, it takes 4-5 minutes

to harvest sufficient energy at 1 meter to initiate the imaging operation. Moreover, following the same analysis discussed above, optimizing the system design parameters would allow reducing this initiation time to few seconds.

### 4.2 Image Framerate

The framerate of our system depends on the time needed to capture and communicate image data to a remote receiver. Specifically, the total time, $T$, needed for one full image is given by the following equation:

$$T = \left(T_{segment} + \frac{\text{Bits}_{\text{segment}}}{\text{Datarate}}\right) \text{Total}_{\text{segments}}$$

Where $T_{segment}$ is the time needed to capture and store an image segment in the FPGA's memory and it is equal to 0.7 seconds (Supplementary Table 1), $\text{Bits}_{\text{segment}}$ is the total number of bits in a segment (25000 bits, which includes the bit-equivalent silent period, see FPGA Control and Logic in *Methods*), Datarate is the bitrate of backscatter communication (recall that we used 1 kbps in our experiments), and $\text{Total}_{\text{segments}}$ is equal to the total number of segments (53 segments) in one full image (see FPGA Control and Logic in *Methods*). For a communication data rate of 1 kbps, it takes 1362.1 seconds (~22.7 mins) to capture a grey-scale image and around 68 min to capture a color image. Note that the image transmission is the most time-consuming part because of the low datarate, and the framerate of the system can be improved by increasing the datarate of communication.

To achieve higher framerate, we successfully experimented with communicating at 5 kbps (with BERs of $10^{-3}$ at 1m). At such datarates, the time needed to capture and communicate a grey-scale image reduces to ~5 mins (~14 mins for the color image). Moreover, higher framerates are achievable by leveraging past work on underwater backscatter node design which has demonstrated throughputs up to 20kbps[2]; using such designs would further reduce the time for a grey-scale image to 1.1 mins (3.4 mins for a color image).

## 5. Cost Analysis

The total cost of fabricating and assembling our underwater batteryless imaging sensor prototype is $353.97 (Supplementary Table 3). The main components of the design are the piezoceramic transducers, camera sensor, FPGA, PCB, and housing. The prototype uses a total of six piezoceramic cylinders: two with a resonance frequency of 17 kHz and four with a resonance frequency of 30 kHz. The total cost of the piezoceramic cylinders is $231.5 (45.7*2 +35*4). The housing of the camera prototype consists of a Telesin dome port which costs $45 and a smaller acrylic dome priced at $11 to encapsulate the active illumination hardware. The IGLOO nano FPGA costs $12.72, the Himax camera sensor costs $9.95, and the total cost of PCB fabrication is $12. The low cost of fabrication of our batteryless prototype - coupled with the fact that it does not require an extensive infrastructure in the form of cabling for power and communication[16,17,18,19] - makes underwater backscatter imaging a viable method for scalable underwater imaging.

## 6. Supplementary Discussion

We discuss the performance of our underwater wireless imaging method in the context of alternative methods for underwater communication.

### 6.1. Comparison to Low-Power Acoustic Modems

Our imaging method leverages acoustic backscatter communication to communicate image data at net-zero power. Our evaluation demonstrates that the method achieves communication ranges that are comparable to state-of-the-art low-power underwater modems, albeit at much lower power. Specifically, a state-of-the-art low-power acoustic modem[20] requires 80 milli-Watts to transmit data at 1 kbps over 100m, while our prototype consumes 59 microwatts to transmit data at the same rate over 40m (see Fig. 4e in Main, and see Backscatter Communication Phase in Table 1 of Supplementary). Our analysis demonstrates that higher ranges are realizable with more optimized transducers (see Range Analysis in *Supplementary Information*).

One might wonder whether prior low-power modems could be operated entirely based on harvested acoustic energy and used for net-zero-power underwater imaging. To answer this question, we consider the amount of time needed to harvest sufficient energy to transmit an image using a state-of-the-art low-power modem. Since the modem operates at the same data rate as our backscatter prototype, it would require the same amount of time for image transmission (1362.1seconds, see Supplementary Table 2) to capture and transmit a grayscale image. Multiplying this by the communication power (80mW) results in 106.07 Joules, which is 594x higher than our backscatter-based wireless platform. If one were to harvest this energy from an acoustic source (which can typically provide a few hundreds of microwatts, see Range Analysis in *Supplemental Information*), it would take 4-6 days to harvest sufficient energy before initiating an imaging operation (in comparison to our power-up time of 10-12 seconds). Thus, it would be impractical to design an underwater battery-free wireless imaging system leveraging prior low-power underwater acoustic modems.

Here, it is worth noting that backscatter communication does not eliminate the energy requirements altogether; instead, it shifts the burden of power consumption from the backscatter node to a remote acoustic source (which could be on a drone, submarine, ship, or coastal base station) with a dedicated power source. According to the range analysis in prior work[7] (see Range Analysis in *Supplementary Information*), acoustic backscatter can operate at distances of hundreds of meters under optimized system design parameters. Hence, with careful engineering design, it would be possible to leverage underwater backscatter sensor nodes for low-cost, scalable undersea observations.

### 6.2. Comparison to Alternative Underwater Communication Technologies

Next, we compare underwater acoustic backscatter to alternative underwater wireless communication modalities that do not leverage acoustic signals[21,22]. Underwater optical communication systems can achieve higher data rates than acoustic communication systems (up to Gbps), but their range is limited by the turbidity of water[21]. Specifically, the communication range of low-power optical modems is less than 10 meters in turbid waters[23,24], in contrast to hundreds of meters (or kilometers) for underwater acoustic communications[21] (including

underwater acoustic backscatter[7]). Aside from optical communications, some underwater communication systems use radio frequency signals, such as very-low-frequency (VLF) and extremely-low-frequency (ELF); these systems can achieve underwater communication up to few kilometers, but require kilometer-long antennas[25], which makes them bulky, expensive, and impractical for a compact underwater imaging system. Finally, researchers have considered higher-frequency RF communication technologies for underwater communication (such as Bluetooth or WiFi), but these are limited to a few tens of centimeters of range[26], making them undesirable for underwater communication.

In summary, our evaluation and analysis demonstrate that acoustic backscatter is a viable approach for low-cost, low-power, and long-range imaging of underwater environments. Fundamentally, the power asymmetry inherent to acoustic backscatter communication makes it a desirable approach for underwater sensor nodes, and enables the design of batteryless underwater cameras that could be used for long-term sustainable sensing of the underwater world.

## 7. Supplementary Figures

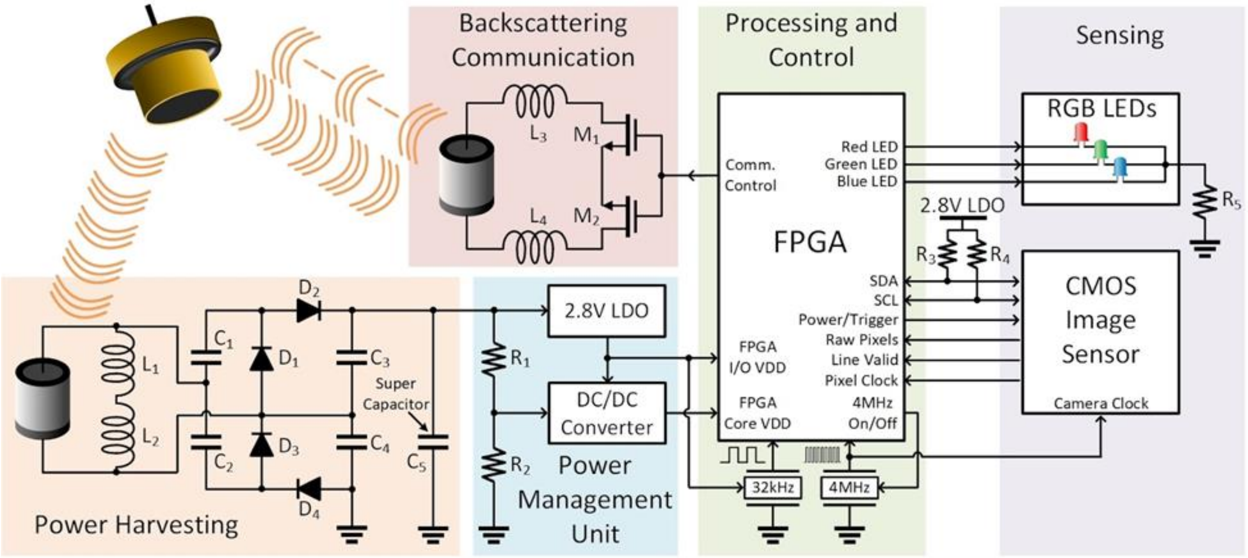


**Fig. 1: Schematic of the hardware design.** The harvester node at the bottom is connected to a multi-stage rectifier followed by a supercapacitor, which stores the harvested energy. The supercapacitor voltage is fed to a 2.8V LDO and to a 1.4V DC/DC step-down converter. The output of the DC/DC converter is used to power the FPGA core, and the output of the LDO is used to power the FPGA banks. The FPGA is also connected to two external clocks (32kHz and 4MHz) and to the camera via several GPIO pins (pixel clock, line valid, data, power, master clock). The FPGA controls the operation of the MOSFETs connected to the communication transducer on the top left. This transducer is responsible for sending camera data via backscatter communication.

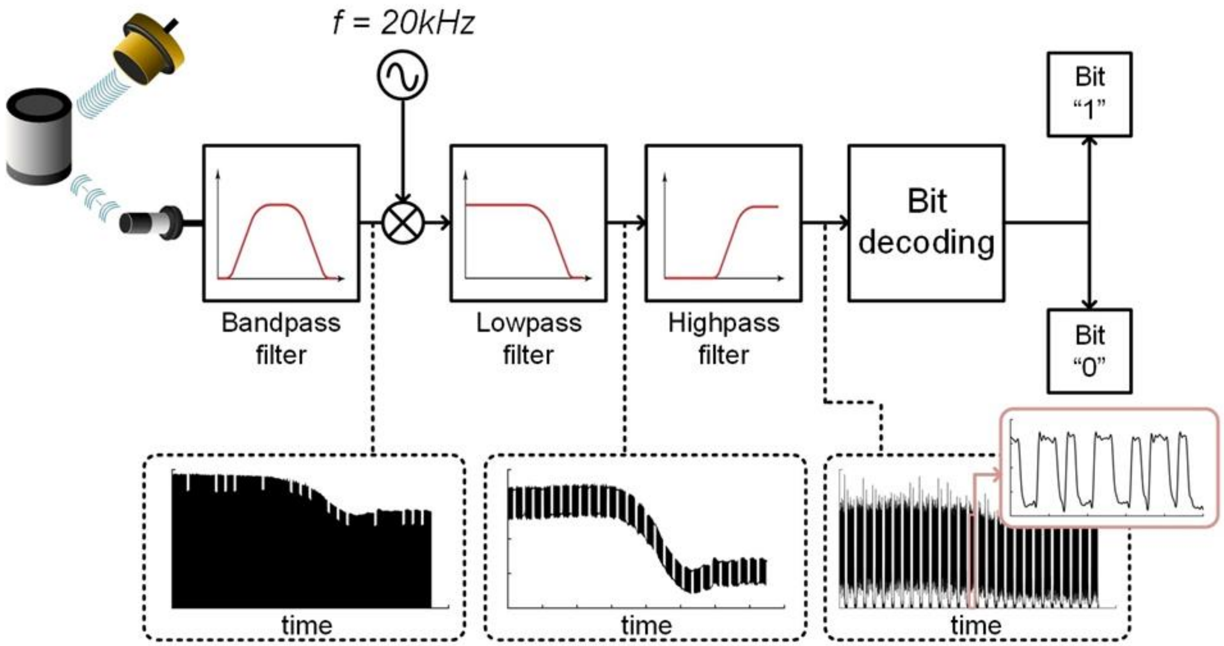


**Fig. 2**: **Demodulation and decoding pipeline.** The signal received by the hydrophone is passed through a band-pass filter, then downconverted and passed through a low-pass filter to remove noise. This signal is then passed through a high-pass filter to remove the signal variations caused by low-frequency surface waves. The demodulated and filtered signal is fed to a maximum likelihood decoder.

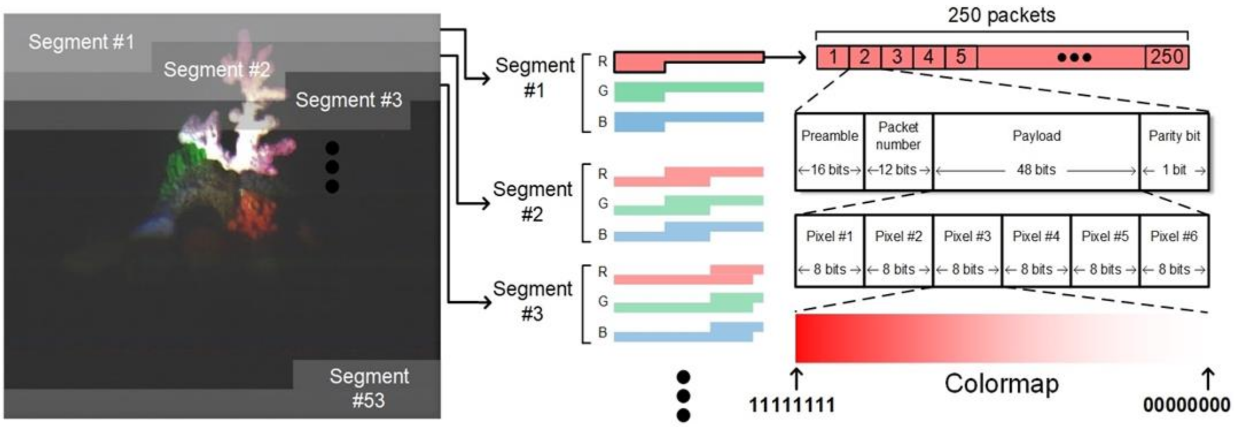


**Fig. 3: Packetization of pixel data.** The image captured by the CMOS image sensor is divided into 53 segments. Each image segment is divided into 250 packets, where each packet contains data for 6 pixels. The uplink packet structure includes a 16-bit preamble, followed by a 12-bit long packet number, and a payload of 48 bits. A parity bit is appended to each packet; it is set to 1 if the sum of bits in the payload is even and is set to 0 otherwise.

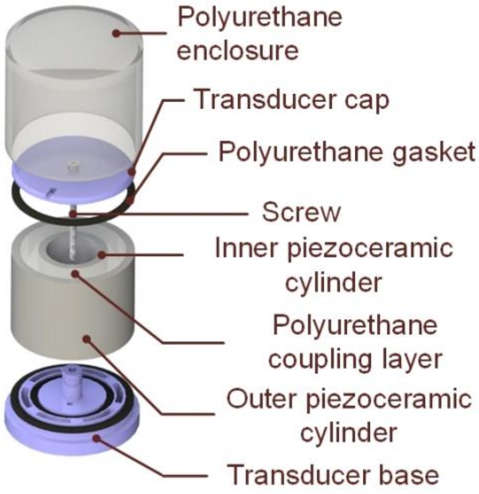


**Fig. 4**: **Exploded view of the layered transducer.** The structure contains a polyurethane layer which is sandwiched between piezoceramic cylinders. The outer piezoceramic cylinder has a nominal resonance frequency of 17kHz, while the inner piezoceramic cylinder has a nominal resonance frequency of 30 kHz. Top and base caps are padded with polyurethane gaskets, and the entire structure is tightened with a screw, then encapsulated with another layer of polyurethane.

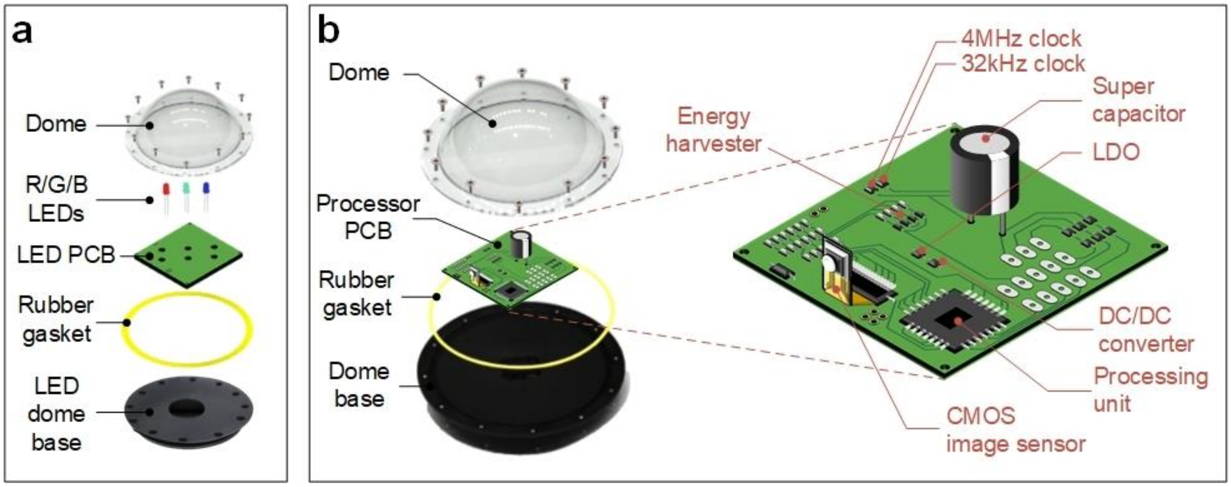


**Fig. 5**: **Exploded view of the camera dome and LED dome.** (**a**) The LED dome contains the red (R), green (G), and blue (B) LEDs, and a layer of polyurethane gasket is added to the dome base to make it water-proof. (**b**) The camera PCB contains the Himax image sensor, supercapacitor for harvesting energy, power management electronics, and an FPGA for processing and memory. It also contains programming pins to program the FPGA and change camera parameters. The PCB is enclosed in a transparent dome, and the entire structure is tightly screwed to make it water-proof.

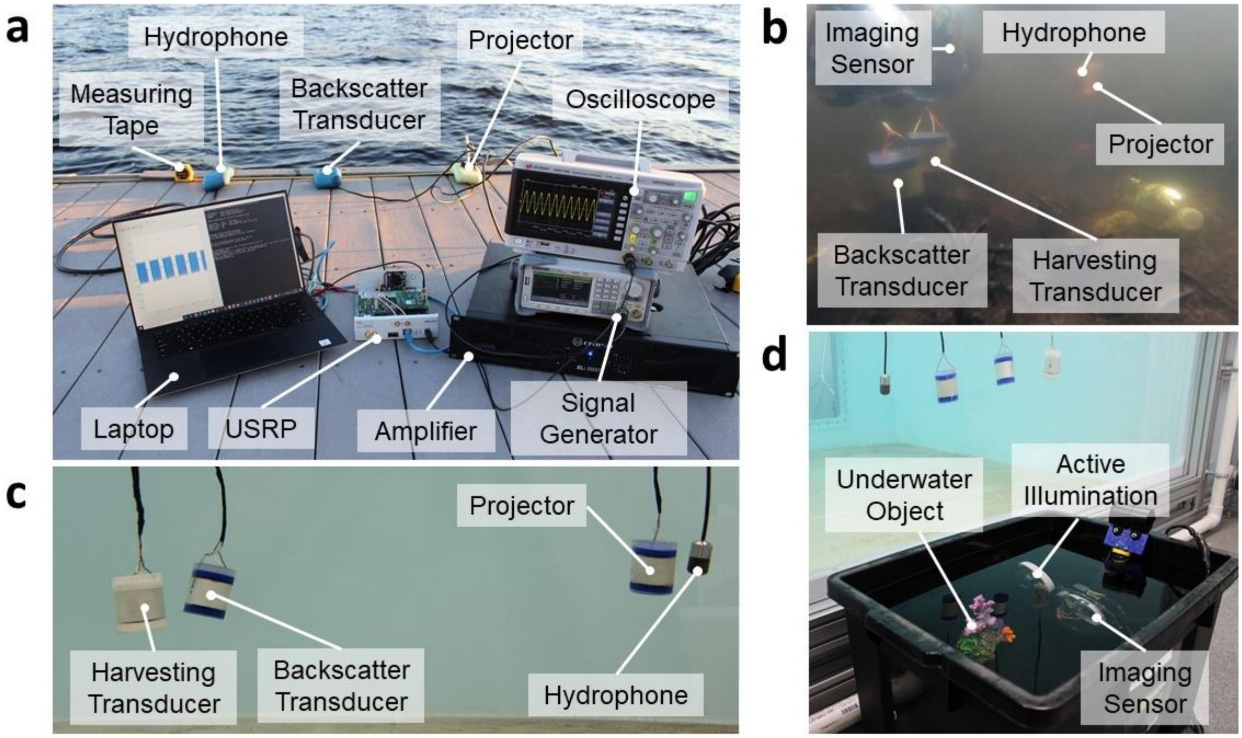


**Fig. 6**: **The prototype evaluation in enclosed and open environments.** (**a**) shows the experimental setup in Charles River, MA. (**b**) shows the underwater setup in Keyser Pond, NH. (**c**) shows the nodes placed in the larger enclosed tank in the lab. (**d**) shows the experimental setup while imaging in the smaller external tank.

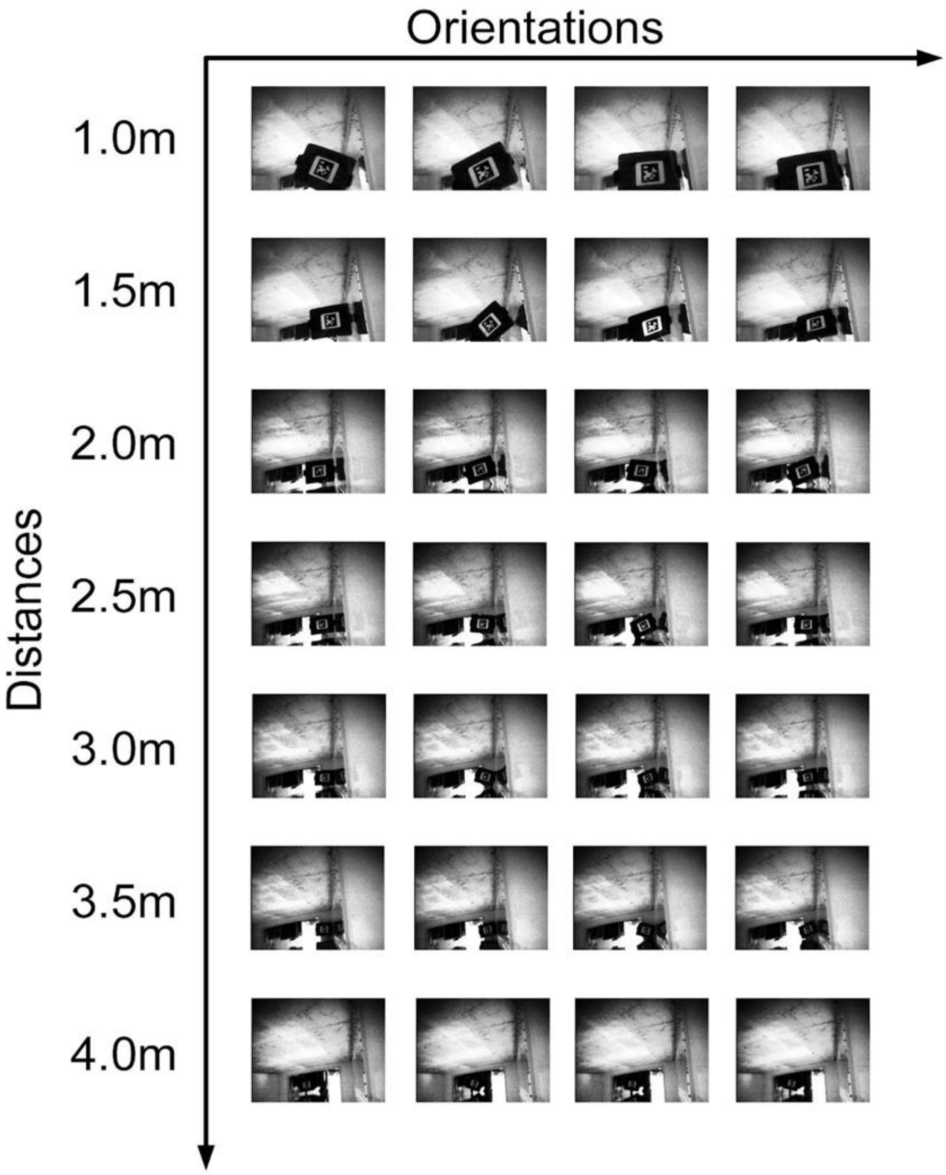


**Fig. 7: Sample AprilTag images.** The camera prototype was used to capture a total of 160 AprilTag images at different distances, orientations, and angles.

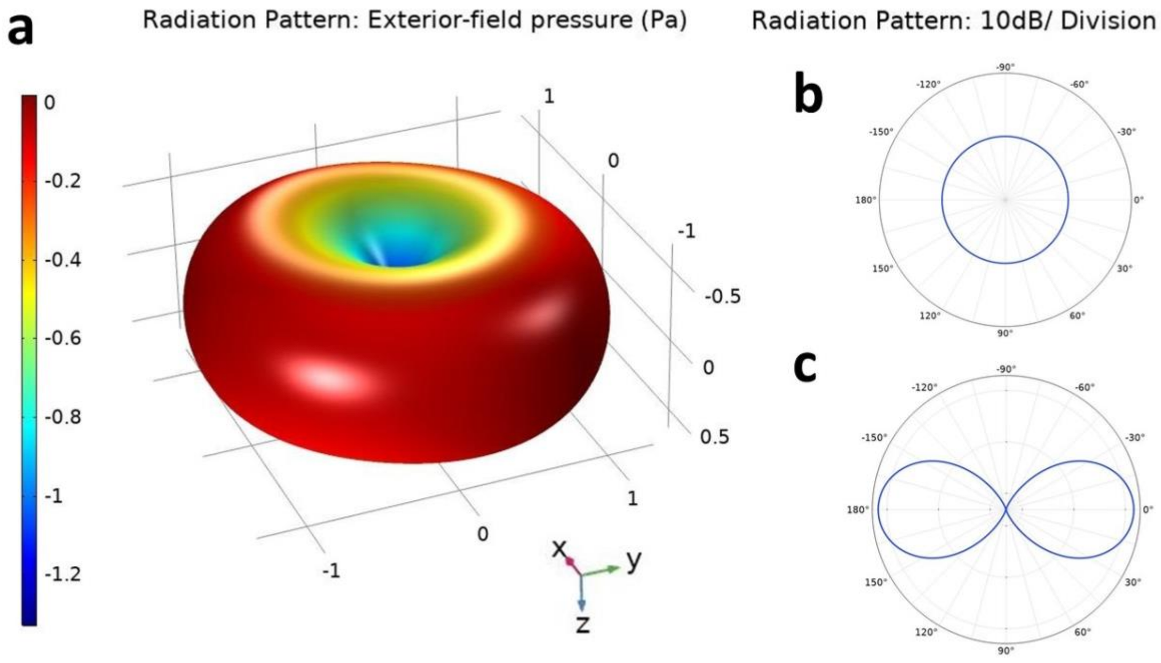


**Fig. 8: Directivity of the layered transducer.** (**a**) shows the pressure radiation heatmap of the layered transducer obtained using COMSOL Multiphysics software. Dark blue regions correspond to low pressure, while dark red regions represent higher pressure. The layered transducer has a directivity index of 2.62 dB (**b**) shows the transverse cut of the radiation pattern which demonstrates that the layered transducer is omnidirectional in the horizontal plane. (**c**) shows the lateral cut of the radiation pattern.

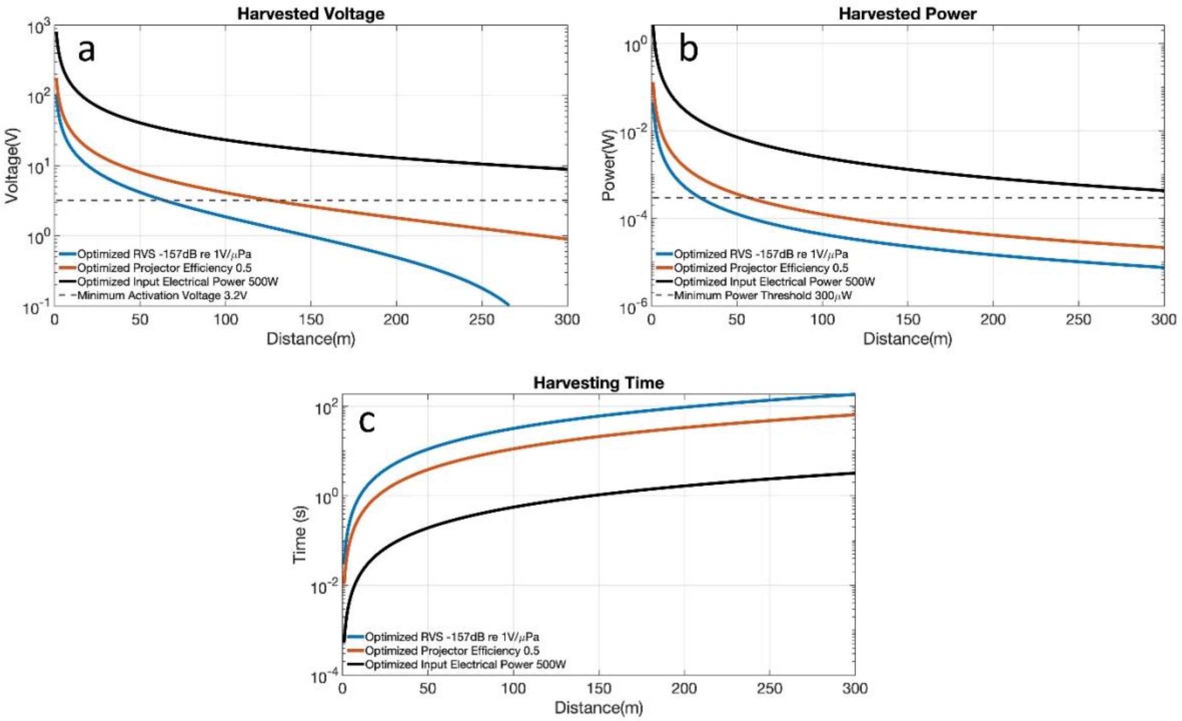


**Fig. 9: Range analysis for the camera prototype.** (**a**) shows the rectified voltage as a function of the distance between the transmitter and the battery-free camera prototype. (**b**) shows the harvested electrical power plotted as a function of the distance between the transmitter and the battery-less camera prototype. (**c**) shows the harvesting time as a function of distance.

## 8. Supplementary Tables

**Table 1: Power consumption for active color imaging**.

| | | **Image Capture Phase** (700 ms per image segment) | | **Backscatter Communication Phase** (25 s per image segment) | |
|---|---|---|---|---|---|
| # | **Device Component** | **Power Consumption (mW)** | **Energy Consumption (mJ)** | **Power Consumption (mW)** | **Energy Consumption (mJ)** |
| 1 | Camera Sensor | 1.1 | 0.77 | 0 | 0 |
| 2 | Active Illumination (R,G,B) | (8.22696,4.391,2.71) | (5.7588,3.0737, 1.8977) | 0 | 0 |
| 3 | AGLN060 FPGA | 0.4807 | 0.336 | 0.0224 | 0.56 |
| 4 | 4MHz oscillator | 0.168 | 0.1176 | 0 | 0 |
| 5 | 32kHz oscillator | 0.0126 | 0.0088 | 0.0126 | 0.3150 |
| 6 | DC-DC Step Down Converter | 0.054867 | 0.0384 | 0.001244 | 0.0311 |
| 7 | Low dropout (R,G,B) | (1.4372, 1.3127, 0.575233) | (1.00604, 0.91889, 0.4026631) | 0.022854 | 0.57135 |
| 8 | N-Channel MOSFETs | 0 | 0 | 24e-9 | 6e-7 |
| | **Average Power Consumption** | [Total Energy Consumption (Image Capture Phase) + Total Energy Consumption (Backscatter Communication Phase)] / Total time = [894.2 mJ + 234.91 mJ]/4086.3 s = 0.276 mW = **276.31 μW** | | | |

The table shows the power consumption breakdown for each component in the prototype while performing active imaging. The energy consumption is computed and shown separately for each of the image capture and backscatter communication phases. Since there are 53 segments per image and each segment is repeated three times (once for each active illumination), the average power consumption of capturing and communicating an entire color image is 276.31 μW.

**Table 2: Power consumption for passive grayscale imaging**.

| | | **Image Capture Phase** (700 ms per image segment) | | **Backscatter Communication Phase** (25 s per image segment) | |
|---|---|---|---|---|---|
| # | **Device Component** | **Power Consumption (mW)** | **Energy Consumption (mJ)** | **Power Consumption (mW)** | **Energy Consumption (mJ)** |
| 1 | Camera Sensor | 1.1 | 0.77 | 0 | 0 |
| 2 | AGLN060 FPGA | 0.4807 | 0.33649 | 0.0224 | 0.56 |
| 3 | 4MHz oscillator | 0.168 | 0.1176 | 0 | 0 |
| 4 | 32kHz oscillator | 0.0126 | 0.0088 | 0.0126 | 0.3150 |
| 5 | DC-DC Step Down Converter | 0.054867 | 0.0384 | 0.001244 | 0.0311 |
| 6 | Low dropout | 0.1848 | 0.12936 | 0.022854 | 0.57135 |
| 7 | N-Channel MOSFETs | 0 | 0 | 24e-9 | 6e-7 |
| | **Average Power Consumption** | [Total Energy Consumption (Image Capture Phase) + Total Energy Consumption (Backscatter Communication Phase)] / Total time = [74.234 mJ + 78.304 mJ]/1362.1 s = 0.112 mW = **111.98 µW** | | | |

This table shows the power consumption breakdown for each component of the prototype while performing passive grayscale imaging. The energy consumption is computed and shown separately for each of the image capture and backscatter communication phases. The average power consumption of capturing and communicating an entire grayscale image is 111.98µW.

**Table 3: Cost breakdown of battery-free underwater camera prototype.**

| # | Device Component | Quantity | Cost ($) |
|---|---|---|---|
| 1 | Piezo Ceramic Cylinder (17 kHz) | 2 | 91.50 |
| 2 | Piezo Ceramic Cylinder (30 kHz) | 4 | 140.00 |
| 3 | Polyurethane Elastomer WC-575 A/B | 1 (0.03 Gallon) | 4.34 |
| 4 | HiMax HM01B0 Camera Sensor | 1 | 9.95 |
| 5 | IGLOO nano AGLN060 FPGA | 1 | 12.72 |
| 6 | TELESIN 6” Dome Port | 1 | 45.00 |
| 7 | SupremeTech Acrylic 3’’ Dome Hemisphere | 1 | 10.99 |
| 8 | PCB Fabrication | 1 | 12.00 |
| 9 | Inductors HM3341ND | 6 | 18.98 |
| 10 | Electrical Components (including Oscillators, Capacitors, Resistors, Diodes) | - | 19.48 |
| | | **Total Cost** | **$353.97** |

This table shows the cost breakdown of the underwater battery free imaging prototype. The overall cost of building a battery-free imaging sensor is $353.97.

## 9. Supplementary References


1. Jang, J., & Adib, F. (2019). Underwater backscatter networking. In *Proceedings of the ACM Special Interest Group on Data Communication* (pp. 187-199).
2. Ghaffarivardavagh, R., Afzal, S. S., Rodriguez, O., & Adib, F. (2020, July). Ultra-wideband underwater backscatter via piezoelectric metamaterials. In *Proceedings of the Annual conference of the ACM Special Interest Group on Data Communication on the applications, technologies, architectures, and protocols for computer communication* (pp. 722-734).
3. Forsyth, D., Ponce, J., Mukherjee, S., & Bhattacharjee, A. K. (2012). Computer vision: a modern approach (Vol. 2). Cambridge: Pearson.
4. Zhang, Z. (2000). A flexible new technique for camera calibration. IEEE Transactions on pattern analysis and machine intelligence, 22(11), 1330-1334.
5. Olson, E. (2011, May). AprilTag: A robust and flexible visual fiducial system. In 2011 IEEE International Conference on Robotics and Automation (pp. 3400-3407). IEEE.
6. Roman, J. et al. (2013). The Marine Mammal Protection Act at 40: status, recovery, and future of US marine mammals. Annals of the New York Academy of Sciences, 1286(1), 29-49.
7. Bereketli, Alper. "Interference-Free Source Deployment for Coverage in Underwater Acoustic Backscatter Networks." Peer-to-Peer Networking and Applications (2022): 1-18.
8. Bereketli, Alper, and Semih Bilgen. "Remotely powered underwater acoustic sensor networks." IEEE Sensors Journal 12.12 (2012): 3467-3472.
9. Sherman, Charles H., and John L. Butler. Transducers and arrays for underwater sound. Vol. 4. New York: Springer, 2007.
10. Kuphaldt, Tony. "Lessons in electric circuits, volume iii–semiconductors." (2009).
11. H. Cha, W. Park and M. Je, "A CMOS Rectifier With a Cross-Coupled Latched Comparator for Wireless Power Transfer in Biomedical Applications," in *IEEE Transactions on Circuits and Systems II: Express Briefs*, vol. 59, no. 7, pp. 409-413, July 2012, doi: 10.1109/TCSII.2012.2198977.
12. Tabesh, Ahmadreza, and Luc G. Fréchette. "A low-power stand-alone adaptive circuit for harvesting energy from a piezoelectric micropower generator." *IEEE Transactions on Industrial Electronics* 57.3 (2009): 840-849.
13. Wang, Yong, et al. "Robust high-order superdirectivity of circular sensor arrays." *The Journal of the Acoustical Society of America* 136.4 (2014): 1712-1724.
14. Seo, Dongjin, et al. "Ultrasonic beamforming system for interrogating multiple implantable sensors." 2015 37th Annual International Conference of the IEEE Engineering in Medicine and Biology Society (EMBC). IEEE, 2015.
15. Hassanieh, Haitham, et al. "Fast millimeter wave beam alignment." *Proceedings of the 2018 Conference of the ACM Special Interest Group on Data Communication*. 2018.
16. Katija, K., Sherlock, R. E., Sherman, A. D., & Robison, B. H. (2017). New technology reveals the role of giant larvaceans in oceanic carbon cycling. *Science Advances, 3*(5), e1602374.

17. Robison, B. H., Reisenbichler, K. R., & Sherlock, R. E. (2017). The coevolution of midwater research and ROV technology at MBARI. *Oceanography*, *30*(4), 26-37.
18. Wiebe, P. H., & Benfield, M. C. (2003). From the Hensen net toward four-dimensional biological oceanography. *Progress in Oceanography*, *56*(1), 7-136.
19. Lam, K., Bradbeer, R. S., Shin, P. K., Ku, K. K. & Hodgson P. , Application of a real-time underwater surveillance camera in monitoring of fish assemblages on a shallow coral communities in a marine park in OCEANS 2007. 1-7 (IEEE).
20. Sanchez, A., Blanc, S., Yuste, P., & Serrano, J. J. (2011, June). A low cost and high efficient acoustic modem for underwater sensor networks. In OCEANS 2011 IEEE-Spain (pp. 1-10). IEEE.
21. Schirripa Spagnolo, Giuseppe, Lorenzo Cozzella, and Fabio Leccese. "Underwater optical wireless communications: Overview." *Sensors* 20.8 (2020): 2261.
22. Gussen, Camila MG, et al. "A survey of underwater wireless communication technologies." J. Commun. Inf. Sys 31.1 (2016): 242-255.
23. Oubei, Hassan Makine, et al. "2.3 Gbit/s underwater wireless optical communications using directly modulated 520 nm laser diode." Optics express 23.16 (2015): 20743-20748.
24. Elamassie, Mohammed, Farshad Miramirkhani, and Murat Uysal. "Performance characterization of underwater visible light communication." IEEE Transactions on Communications 67.1 (2018): 543-552.
25. Lanzagorta, Marco. "Underwater communications." *Synthesis lectures on communications* 5.2 (2012): 1-129.
26. Strama, Kay, Daniel Weber, and Helge Renkewitz. "Evaluation of Wifi data transmission algorithms for short distance underwater communication." OCEANS 2021: San Diego–Porto. IEEE, 2021.
