# Supplementary material for: Battery-free wireless imaging of underwater environments
Source: Nat Commun. 2022 Sep 26;13:5546. doi: 10.1038/s41467-022-33223-x (PMC9512789; doi:10.1038/s41467-022-33223-x)
Supplement: Supplementary file 2 — Description of Additional Supplementary Files [file 41467_2022_33223_MOESM2_ESM.pdf]

Description of Additional Supplementary Files

Supplementary Movie 1:

To the right, we see an underwater measurement setup with a batteryless sensor and active illumination. The setup is imaging an underwater object, which is a coral model in this experiment. To the left, we see the color image that is being received and reconstructed. The method starts using red active illumination, followed by transmitting an image segment that is received and shown to the left. The image segment is received in packets and applied to the red channel. Once the first red segment is completed, the method uses green active illumination to capture and send the same image segment, which is applied to the green channel of the color image at the receiver. Subsequently, the same is repeated using blue active illumination, which is again received in packets and applied to the blue channel of the received image. The same process repeats for other segments of the image, allowing full reconstruction of color images at the receiver as can be seen to the left.
